# Structure of an Inner Membrane Protein Required for PhoPQ-Regulated Increases in Outer Membrane Cardiolipin

**DOI:** 10.1128/mBio.03277-19

**Published:** 2020-02-11

**Authors:** Junping Fan, Erik M. Petersen, Thomas R. Hinds, Ning Zheng, Samuel I. Miller

**Affiliations:** a Department of Microbiology, University of Washington, Seattle, Washington, USA; b Howard Hughes Medical Institute, Department of Pharmacology, University of Washington, Seattle, Washington, USA; c Department of Genome Sciences, University of Washington, Seattle, Washington, USA; d Department of Medicine, University of Washington, Seattle, Washington, USA; Yale University School of Medicine

**Keywords:** PbgA, CL transport, PhoPQ, outer membrane barrier, salmonellae

## Abstract

The Salmonella enterica subsp. enterica serovar Typhimurium PhoPQ two-component system is activated within the intracellular phagosome environment, where it promotes remodeling of the outer membrane and resistance to innate immune antimicrobial peptides. Maintenance of the PhoPQ-regulated outer membrane barrier requires PbgA, an inner membrane protein with a transmembrane domain essential for growth, and a periplasmic domain required for PhoPQ-activated increases in outer membrane cardiolipin. Here, we report the crystal structure of cardiolipin-bound PbgA, adopting a novel transmembrane fold that features a cardiolipin binding site in close proximity to a long and deep cleft spanning the lipid bilayer. The end of the cleft extends into the periplasmic domain of the protein, which is structurally coupled to the transmembrane domain via a functionally critical C-terminal helix. In conjunction with a conserved putative catalytic dyad situated at the middle of the cleft, our structural and mutational analyses suggest that PbgA is a multifunction membrane protein that mediates cardiolipin transport, a function essential for growth, and perhaps catalysis of an unknown enzymatic reaction.

## Abstract (precis)

Gram-negative bacteria cause many types of infections and have become increasingly resistant to available antibiotic drugs. The outer membrane serves as an important barrier that protects bacteria against antibiotics and other toxic compounds. This outer membrane barrier function is regulated when bacteria are in host environments, and the protein PbgA contributes significantly to this increased barrier function by transporting cardiolipin to the outer membrane. We determined the crystal structure of PbgA in complex with cardiolipin and propose a model for its function. Knowledge of the mechanisms of outer membrane assembly and integrity can greatly contribute to the development of new and effective antibiotics, and this structural information may be useful in this regard.

## INTRODUCTION

The Gram-negative bacterial cell envelope consists of two lipid bilayers, an inner membrane (IM) and an outer membrane (OM), separated by a periplasmic space which contains a rigid layer of peptidoglycan (1). Unlike the symmetric IM, the OM is largely asymmetric, with the outer leaflet composed predominately of lipopolysaccharide (LPS) and an inner leaflet consisting of glycerophospholipids (GPL) (2–4). The asymmetry of the OM serves as a selective barrier, permitting the uptake of necessary nutrients while blocking the entry of toxic compounds. The OM is a complex organelle containing pore-forming β-sheet membrane proteins that contribute to the selective barrier (5, 6), multiprotein organelles, including secretion machineries and flagella that bridge the membranes (7, 8), and lipoproteins that can form a covalent link from peptidoglycan to the OM (9). The barrier activity of the OM is regulated by environmental conditions, enabling the bacteria to respond to rapidly changing conditions, such as those found in host tissues (10). This regulation is important for bacteria to resist killing by toxic compounds and is also essential to microbial pathogenesis for animals (11, 12).

In salmonellae and other enteric bacteria, the PhoPQ two-component system plays a critical role in environmental sensing to regulate the OM barrier (13). Salmonella enterica subsp. enterica serovar Typhimurium (S. Typhimurium) is able to invade and replicate within macrophages where the bacterium remains within a phagocytic vacuole (14). Both the reduction of pH within this vacuole and the presence of host antimicrobial peptides activate the PhoQ IM sensor kinase, stimulating phosphorylation of the PhoP transcriptional regulator (15). PhoPQ regulates all classes of OM components, including the protein content, LPS structure, and GPL composition within the lipid bilayer (16). The activation of PhoPQ triggers LPS acylation and charge modifications of LPS phosphates, which result in a more hydrophobic surface. PhoPQ activation also leads to an increase in the acylation of phosphatidylglycerol and an increase in cardiolipin (CL) within the OM, which should further increase its hydrophobicity (17). A recent genetic screen identified an IM protein, termed PhoPQ barrier gene A (PbgA), as crucial for PhoPQ-regulated CL transport to the OM and maintenance of OM barrier function when PhoPQ is activated (18). PbgA has a five-transmembrane (TM) N-terminal IM domain that is essential for growth *in vitro* within rich medium (19). It also contains a large periplasmic domain required for transporting CL to the OM and maintaining OM barrier function when PhoPQ is activated. The PbgA periplasmic domain function of delivering acidic phospholipids to the OM is essential for *S.* Typhimurium infection, as partial deletion of it leads to highly attenuated microbial virulence and survival within macrophages (18).

Previous studies have reported the structures of the soluble periplasmic domains of *S.* Typhimurium PbgA and its Escherichia coli homologue YejM (20). Dong et al. showed that the PbgA periplasmic domain structure has a fold similar to that of other members of the arylsulfatase protein family, though the typical enzymatic site of this domain was not predicted to be active. Dong et al. also identified a hydrophobic pocket that may be responsible for binding and transporting CL. However, they did not define the structure in the presence of bound CL, and the orientation of the periplasmic domain to the membrane was unknown since their structure did not contain the N-terminal TM domain (20). Thus, full-length structural information that could impact how the PbgA periplasmic domain acts together with the TM domain to mediate CL transport was unknown. We determined the crystal structure of the full-length *S.* Typhimurium PbgA protein in complex with CL at 2.7-Å resolution to provide a molecular structural foundation to understand PbgA function. This structure not only unveils a unique architecture of PbgA with two CL-binding sites, but also a long and deep membrane-penetrating cleft harboring an unexpected putative catalytic dyad.

## RESULTS

### The full-length PbgA structure reveals a novel structural fold.

The PbgA coding sequence was cloned into the pBAD24 vector and used to overexpress the protein in E. coli DH5α. When the recombinant protein was subjected to trypsin digestion, a major truncated product with a size equal to that of the PbgA periplasmic domain was identified and further confirmed by matrix-assisted laser desorption ionization–time of flight (MALDI-TOF) mass spectrometry analysis (see Fig. S1 in the supplemental material). This result is consistent with the predicted flexibility of the region connecting the TM region and periplasmic domain. We subsequently subcloned and purified the periplasmic domain of PbgA (PbgA_PD 245–586) and determined its structure at 1.7-Å resolution using the seleno-based single-wavelength anomalous dispersion (SAD) method. Despite protein stability problems, well-diffracting crystals of the recombinant full-length PbgA protein were later obtained by premixing the protein with CL. The crystal structure of the full-length protein was determined by the molecular replacement method using PbgA_PD as the search model at 2.7-Å resolution. The crystal form belongs to the P2_1_ space group and contains two protein molecules per asymmetric unit. The two monomers of PbgA pack in a head-to-head fashion and can be superimposed with a root mean square deviation of 1.043 Å. Size-exclusion chromatography with multiangle light scattering (SEC-MALS) analysis revealed that PbgA was monomeric in solution. The structure model was refined to an *R*_work_ value of 23.24% and *R*_free_ value of 29.02% (Table S1).

10.1128/mBio.03277-19.1 FIG S1 Proteolytic digestion of purified PbgA by trypsin. Representative SDS-PAGE patterns of time course trypsin digestion of PbgA are shown. Digestion was stopped at the times indicated by horizontal numbers. No trypsin was added to the control (Ctrl) sample. The bands are labeled based on mass spectrometry results. Download FIG S1, PDF file, 1.2 MB. Copyright © 2020 Fan et al. 2020 Fan et al. This content is distributed under the terms of the Creative Commons Attribution 4.0 International license.

10.1128/mBio.03277-19.2 FIG S2 Helical wheel plot of the PbgA C-terminal helix. This segment has a pronounced amphiphilic character in which most of the hydrophobic residues (yellow) and most of the charged residues (red and blue) are placed on opposite faces of the helix. Download FIG S2, PDF file, 1.1 MB. Copyright © 2020 Fan et al. 2020 Fan et al. This content is distributed under the terms of the Creative Commons Attribution 4.0 International license.

10.1128/mBio.03277-19.7 TABLE S1 Data collection and refinement statistics. Download Table S1, DOCX file, 0.1 MB. Copyright © 2020 Fan et al. 2020 Fan et al. This content is distributed under the terms of the Creative Commons Attribution 4.0 International license.

**FIG 1:**
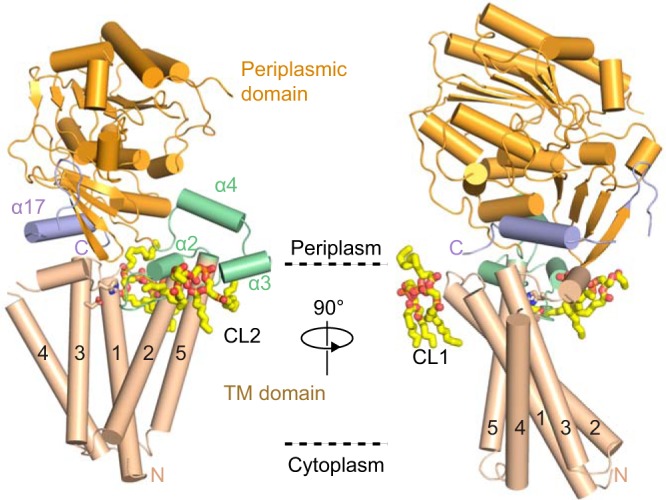
Overall structure of PbgA, with two orthogonal views in a cartoon representation. The TM domain, linker region, periplasmic domain, and C-terminal region are colored in wheat, pale green, bright orange, and light blue, respectively. TM helices are numbered 1 to 5, the α-helices in the linker region are labeled α2 to α4, and the C-terminal region is labeled α17. CL molecules are shown in a stick model and labeled with CL1 for binding site 1 and CL2 for binding site 2. The predicted membrane boundaries are indicated by dashed lines.

A Dali search was performed to further understand the molecular details of PbgA function, which indicated that the TM domain of PbgA represents a novel fold that has not been observed previously. As seen in the structure, PbgA is composed of a five-TM (TMs 1 to 5) helix domain (amino acids [aa] 7 to 190) and a large periplasmic domain (aa 241 to 586) that are connected by a 50-residue linker region (aa 191 to 240) (Fig. 1). The N terminus is located on the cytosolic side, while the C terminus is on the periplasmic side. The TM region of PbgA adopts a tilted and flat core that is formed by TMs 1 to 3 and is surrounded by TMs 4 and 5 in a V-shape pattern. The periplasmic domain is connected to TM5 by a linker region and sits on top of the TM domain. With two layers of β-sheets sandwiched by three layers of helical bundles, this overall fold of the periplasmic domain is similar to the previously reported structure of the isolated PbgA periplasmic domain (20), as well as other structures from the same family of proteins (21). Interestingly, based on sequence similarity, the PbgA periplasmic domain belongs to the alkaline phosphatase superfamily. However, the PbgA periplasmic domain does not contain the catalytic residues typically found in members of this family, which is consistent with former studies (18, 20). Surprisingly, the linker region connecting the TM region and the periplasmic domain are composed of three helices, labeled α2 to α4 (Fig. 1), and adopt a stable structure, which was not expected given its high trypsin sensitivity within the entire PbgA polypeptide. The periplasmic domain packs against the TM domain largely through an amphipathic α-helix α17 (Fig. 1 and S2) formed by the C-terminal sequence of PbgA.

### The structure of the PbgA-CL complex defines two CL-binding sites.

**FIG 2:**
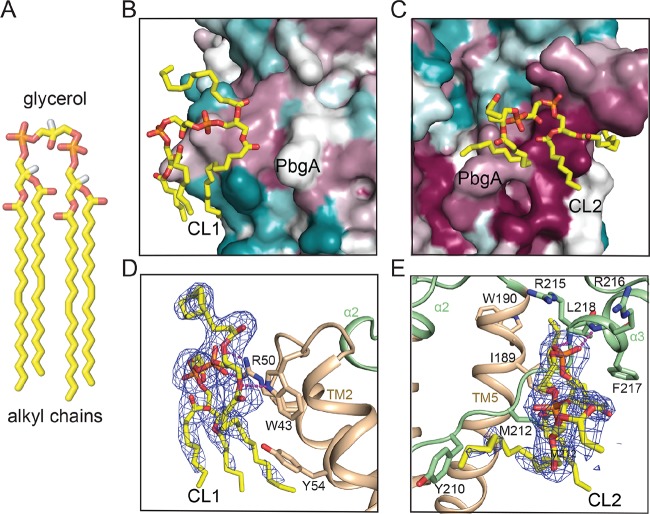
Representation of cardiolipin binding sites. (A) CL structure is displayed in a cartoon model with the alkyl chain tail and glycerol of phosphate-glycerol-phosphate head group labeled. (B and C) Conservation mapping of the two CL-binding sites. PbgA is colored based on sequence conservation, with a gradient from magenta (maximum, 100%) to cyan (minimum, 0%). CL is shown in a stick model and colored by atom, with oxygen in red, phosphate in orange, and carbon in yellow. (D and E) Cartoon representation of CL-binding sites. PbgA is colored as in Fig. 1, with the F_o_-F_c_ map density contoured at 2.0 σ and in blue mesh. Hydrogen bonds and ionic bond are indicated by purple dash lines.

The structural basis of the CL interaction with PbgA was revealed by the addition of CL to the crystal screening (Fig. 2). The electron density map of PbgA revealed two large densities next to the PbgA protein in which CL molecules fit perfectly. We designated the densities CL1 and CL2, which occupied two potential CL-binding sites termed site 1 and site 2, respectively. CL1 is situated in the TM domain, while CL2 is located next to the linker region (Fig. 1 and S3A). We mapped sequence conservation onto the PbgA structure, which revealed further insight (Fig. S3B and S4). Strikingly, in contrast to the low conservation of residues forming site 1 (Fig. 2B), residues at site 2 are highly conserved (Fig. 2C). Each CL has one phosphate of the central phosphate-glycerol-phosphate moiety facing a positive surface area of PbgA (Fig. S3A). For CL1, three of the four alkyl chains are inserted within the membrane portion, while the fourth one is exposed to the periplasm (Fig. 2B). The electrostatic surface of site 1 reveals that one CL1 head group phosphate is bound by PbgA at a positively charged area at the N-terminal end of TM2 (Fig. S3A). A central arginine residue (R50) and the surrounding panel of hydrophobic residues (W43, L47, I51, and Y54) at the TM1-TM2 junction make direct contact with the CL molecule. The indole amine of W43 forms a potential hydrogen bond with one ester linkage of CL1, and ionic bonds were formed between the side-chain amine of R50 to the phosphate moiety on the CL phosphate-glycerol-phosphate head group (Fig. 2D). In addition, hydrophobic interactions are made between the side chains of the PbgA hydrophobic residues and the alkyl chain of CL1. Despite all these interactions, the CL located at site 1 is closely involved in crystal packing (Fig. S5), raising the possibility that it might not be a physiologically relevant CL-binding site.

10.1128/mBio.03277-19.3 FIG S3 Conservation mapping on the PbgA structure. A, surface representation of CL-binding sites. PbgA is colored by electrostatic surface potential mapping with positive in blue, negative in red, and uncharged or hydrophobic in white. CL is shown in stick model and colored as Fig. 1. B, sequence conservation surface mapping of PbgA, which is colored based on sequence conservation with a gradient from magenta (conserved 100%) to cyan (conserved 0%) based on 35 PbgA homologs. Download FIG S3, JPG file, 0.6 MB. Copyright © 2020 Fan et al. 2020 Fan et al. This content is distributed under the terms of the Creative Commons Attribution 4.0 International license.

10.1128/mBio.03277-19.4 FIG S4 Amino acid sequence alignment of PbgA homologs from 10 members of pathogenic Gram-negative bacteria. Identical residues are shown in blue, and conserved residues mentioned in the text are indicated with green triangles. Secondary structure elements are included, with α-helices shown as bars and β-sheets shown as arrows. Dashed lines represent the disordered region in the crystal structure. Download FIG S4, PDF file, 1.5 MB. Copyright © 2020 Fan et al. 2020 Fan et al. This content is distributed under the terms of the Creative Commons Attribution 4.0 International license.

10.1128/mBio.03277-19.5 FIG S5 Crystal packing of PbgA with CL at binding site 1. CL shown in stick binds in the middle of two PbgA molecules shown in surface representation that are found in the asymmetry unit. Download FIG S5, PDF file, 1.7 MB. Copyright © 2020 Fan et al. 2020 Fan et al. This content is distributed under the terms of the Creative Commons Attribution 4.0 International license.

Unlike site 1, the CL molecule bound at site 2 sits at the membrane-periplasm interface, and its alkyl chains are more curved toward PbgA. Remarkably, site 2 is formed almost exclusively by the loop flanked by α2 and α3 of the linker region (Fig. 2C). Analysis of the electrostatic surface of this site revealed that one of the phosphates of the central phosphate-glycerol-phosphate moiety is also facing a positive surface area of PbgA (Fig. S3A). Also, one alkyl chain is inserted into a binding groove formed by the PbgA TM5 and α3 helix (Fig. 2C). Site 2 also presents more CL-interacting residues than does site 1 (residues I189, W190, Y210, M212, R215, R216, F217, and L218). The main-chain amides of residues R215 and R216 form hydrogen bonds with the phosphate moiety, and side chains of residues I189, W190, Y210, M212, F217, and L218 participate in making hydrophobic interactions with the alkyl chains of CL (Fig. 2E). Our laboratory’s previous studies indicated that R215 and R216 are important for CL binding, bacterial growth, and barrier function (18). These findings, together with our crystal structure, strongly support the physiological relevance of CL binding at site 2.

### PbgA contains a long and deep cleft extending from the membrane into the periplasm.

**FIG 3:**
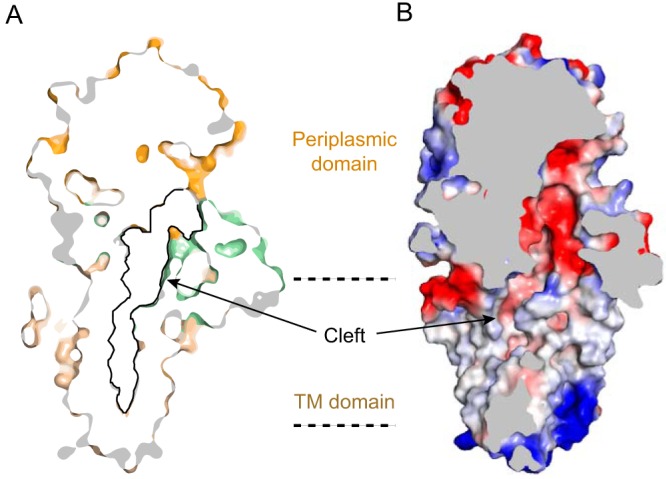
The long and deep cleft of PbgA. (A and B) Sliced surface representation with PbgA colored the same way as described above with a black line denoting the deep cleft (A) or an electrostatic surface representation showing the charge of the cleft (B).

A striking feature of the structure of PbgA is a deep and long cleft on the surface of the protein extending from the TM domain into the periplasmic domain (Fig. 3A). This surface cleft could potentially allow membrane-associated lipid substrates to enter or exit. The surface distribution of electrostatic charges of the PbgA molecule showed that the periplasmic side of the structure is of mixed charges. The cytoplasmic side of the TM domain is highly positively charged, consistent with the “positive-inside rule” (22) that cytosolic loops near the lipid bilayer contain more positively charged amino acids. The interior of the cleft is primarily hydrophobic on the TM domain and is likely exposed to the hydrophobic central region of the lipid bilayer. This area could potentially accept CL acyl chains after its binding to the linker region. Interestingly, the cleft becomes negatively charged within the periplasmic domain (Fig. 3B), suggesting that CL could not easily pass through unless PbgA undergoes significant conformational changes that allow CL to be released.

### The PbgA C terminus is required to maintain PhoPQ-regulated barrier function.

**FIG 4:**
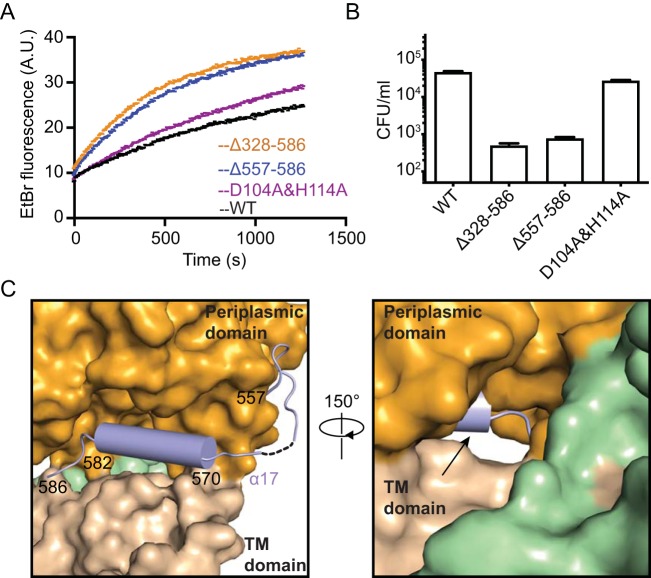
Functional analysis of the PbgA C-terminal region. (A) OM permeability of WT and PbgA mutants measured by ethidium bromide (EtBr) uptake. A.U., arbitrary units. The results are representative of three independent experiments. Deletion of the C-terminal region *pbgA*(Δ557–586) exhibits much higher EtBr uptake than does the WT, mimicking the *pbgA*(Δ328–586) bulk deletion mutant. The mutation of potential catalytic residues Asp104 and His114 (D104A and H114A, respectively) showed a slight difference compared to the WT. (B) Survival of WT and PbgA mutants within BMDMs at 18 h postinfection with BMDM. The results are the averages from three independent experiments with double replicates for each strain. The strain with a deletion of the C-terminal region (Δ557–586 mutant) exhibits decreased intracellular survival similar to that of the previously reported deletion mutant (Δ328–586 strain). The mutation of potential catalytic residues Asp104 and His114 (D104A and H114A, respectively) showed a small difference compared to the WT. (C) Cartoon representation of the C-terminal 30 amino acids shown in light blue. The rest of the structure is colored in wheat for the TM domain and bright orange for periplasmic domain. On the right, the last few amino acids of the C terminus fill the back side of the deepest area of the cleft, indicated by an arrow.

PhoPQ activation promotes remodeling of cell surface components, resulting in an increase in OM barrier function. PbgA, through its N-terminal membrane-spanning domain, also has an unknown essential function to maintain organism viability in addition to its role in increasing barrier function in response to PhoPQ activation. Recent studies indicated that even a partial PbgA periplasmic domain deletion, *pbgA*(Δ328–586), resulted in bacteria with increased OM permeability on PhoPQ activation and decreased virulence (18). In order to further characterize this key region of the protein, we tested different-length variants of PbgA. We tested the functional significance of the extreme C terminus of PbgA by introducing an early stop codon after the sequence encoding Y556 in *pbgA* on the bacterial genome, and OM permeability was measured by ethidium bromide (EtBr) uptake assays (23). The *pbgA*(Δ557–586) mutant strain grew at normal rates in Luria-Bertani (LB) broth medium and showed much higher EtBr uptake than that of the WT and resembled the larger *pbgA*(Δ328–586) mutant strain (Fig. 4A), indicating that the carboxyl terminus is critical to its role in barrier function. The relevance of the last 30 amino acids to virulence was tested by measuring the intracellular survival of the *pbgA*(Δ557–586) mutant strain after phagocytosis by mouse bone marrow-derived macrophages. This mutant strain showed a significant reduction in survival at 18 h postinfection, similar to the previous periplasmic domain *pbgA*(Δ328–586) mutant (Fig. 4B). These results indicate that the PbgA C-terminal 30 amino acids are critical for *S.* Typhimurium macrophage survival and PhoPQ-regulated barrier function. In the structure, the C-terminal 30 amino acids contain residues 557 to 570 that form a flexible loop connecting the C terminus to the rest of the protein, as well as residues 571 to 582 that form an amphipathic helix followed by a short loop consisting of residues 583 to 586 bridging the TM domain and periplasmic domain (Fig. 1 and 4C). Another important feature of this region is the last few amino acids (581 to 584) that fill the back side of the deepest area of the cleft at the TM-periplasmic domain interface (Fig. 4C), and these residues are highly conserved (Fig. S4). Therefore, these structural elements are critical to the PhoPQ-regulated barrier function of PbgA that likely involves the cleft and movement of CL to the OM.

### The PbgA structure contains a putative catalytic site.

**FIG 5:**
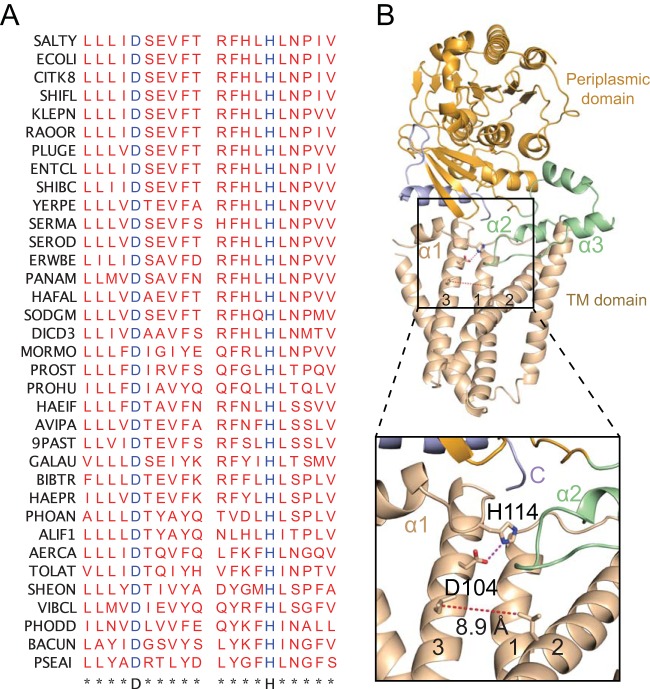
Conserved residues in DUF3413 family form a putative active-site His-Asp dyad. (A) Sequence alignment of 35 DUF3413 family members from Gram-negative bacteria labeled with strain abbreviations and showing the conservation pattern of DUF3413 family in regions surrounding Asp104 and His114. The bacteria used for sequence alignment are *Salmonella* Typhimurium (SALTY), Escherichia coli (ECOLI), Citrobacter koseri (CITK8), Shigella flexneri (SHIFL), Klebsiella pneumoniae (KLEPN), Raoultella ornithinolytica (RAOOR), Pluralibacter gergoviae (PLUGE), Enterobacter cloacae (ENTCL), Shimwellia blattae (SHIBC), Yersinia pestis (YERPE), Serratia marcescens (SERMA), Serratia odorifera (SEROD), Erwinia billingiae (ERWBE), Pantoea ananatis (PANAM), Hafnia alvei (HAFAL), Sodalis glossinidius (SODGM), Dickeya dadantii (DICD3), Morganella morganii (MORMO), Providencia stuartii (PROST), Proteus hauseri (PROHU), Avibacterium paragallinarum (AVIPA), Pasteurella bettyae (9PAST), Gallibacterium anatis (GALAU), Bibersteinia trehalosi (BIBTR), Haemophilus parasuis (HAEPR), Photobacterium angustum (PHOAN), Aliivibrio fischeri (ALIF1), Aeromonas caviae (AERCA), Tolumonas auensis (TOLAT), Shewanella oneidensis (SHEON), Vibrio cholerae (VIBCL), Photobacterium damselae (PHODD), Bacteroides uniformis (BACUN), and Pseudomonas aeruginosa (PSEAI). (B) Cartoon representing of the His-Asp interaction. On the top, PbgA is displayed in cartoon representation and color-coded as in Fig. 1. Putative catalytic dyad residue side chains are displayed as sticks, with nitrogen in blue, oxygen in red, and carbon in yellow. On the bottom is a zoomed-in view of the His-Asp interaction in the cartoon model. A hydrogen bond between D104 and H114 is indicated by a purple dashed line. The red dashed lines show the distance of the V-shaped opening formed by TM2 and TM3.

PbgA orthologs are widely distributed in nature, being not only found in Gram-negative bacteria (24) but also Mycobacterium tuberculosis and the fungus Beauveria bassiana. Despite this conservation among different organisms, its specific function is poorly understood. In order to obtain a better understanding about the functional role of the PbgA family, bioinformatics analysis was performed to evaluate highly conserved residues and potential functional domains. Based on sequence analysis, the PbgA TM region belongs to the domain of unknown function (DUF) 3413 family (25). According to the September 2019 release of the UniProt database and the European Bioinformatics Institute (EMBL-EBI) release from May 2019, the DUF3413 family (Pfam family PF11893) contains 4,237 sequences and 562 sequences, respectively. Interestingly, members of DUF3413 family are found in many multidrug-resistant (MDR) Gram-negative bacteria, including Escherichia coli, Klebsiella pneumoniae, Pseudomonas aeruginosa, *Salmonella* spp., and Neisseria spp. The relationship between DUF3413 proteins in different bacteria was examined by sequence alignment. Despite the low homology (ca. 25% identity) between some of these proteins, we were able to identify multiple conserved residues. We noticed that within the defined ∼250 amino acids of DUF3413, there is a conserved D-X9-H motif found at D104 and H114 in the *S.* Typhimurium PbgA sequence (Fig. 5A andS4). After mapping these residues onto the PbgA structure, we observed the spatial proximity between them (Fig. 5B). These two residues directly interact with each other in a fashion similar to that found in the catalytic centers of various enzymes such as phospholipases (26), suggesting that they might represent a catalytic site. This putative active site is located at the membrane-periplasm interface region and is uncharged. The aspartate and histidine residues are located at TM3 and the loop between TM3 and α1, respectively (Fig. 5B and S4). Moreover, TM2 and TM3 form a V-shaped surface cleft with the periplasmic opening side over 9 Å (Fig. 5B). This opening is situated close to the putative catalytic site and could be involved in substrate binding. Based on sequence analyses and TM helix predictions, all known PbgA homologs are likely to share the same folding topology with PbgA. The two conserved residues, Asp and His, are similarly predicted to interact in their three-dimensional (3D) structures (Fig. 5B and S4). Overall, the crystal structure of PbgA hints at an enzymatic activity that not only might be shared among PbgA homologs but also members of the DUF3413 family. Interestingly, in contrast to truncation of the last 30 amino acids, missense mutations of the two conserved aspartate and histidine residues in the putative catalytic site showed only a small effect on EtBr uptake (Fig. 4A), indicating that these residues contribute only a minor amount to OM barrier function and bacterial survival in BMDM (Fig. 4B), suggesting that these residues and their potential enzymatic activity do not predominately play a role in OM barrier function. Further, our ability to generate this mutant suggests that this potential enzymatic activity is not required for the essential nature of the TM domain. Although there is little evidence that these residues play a role in the function of PbgA, their significant evolutionary conservation suggests a biological role, even if it is only structural in nature. Therefore, given the small effect on barrier function, it is interesting to speculate that this putative catalytic site may function under certain conditions, perhaps in a regulatory capacity on cardiolipin transport.

## DISCUSSION

Here, we report the crystal structure of PbgA, which offers insights into the structure-function relationships of this IM protein found in many Gram-negative bacterial species. According to a Dali search, the overall structure unveils a unique fold, with five TM helices forming the TM domain and a sulfatase-like periplasmic domain connected by a linker region. Furthermore, CL binding to PbgA is facilitated by two arginine residues, Arg215 and Arg216, located in the linker region. Trypsin digestion indicates that the linker region is relatively dynamic, leading us to hypothesize that the CL-bound PbgA structure presented in this paper most likely represents one of several conformations that PbgA adopts.

Previous work demonstrated PbgA serves as a transporter for CL, and here, we present a working model for this process. CL is extracted from the IM by the linker region, which undergoes conformational changes that result in the loading of CL into the cleft of PbgA. This long and deep cleft spanning the whole protein has prerequisite features for transport function. We speculate that CL enters the cleft of PbgA on the TM side and exits through the periplasmic side. The periplasmic side would then undergo topologic changes in order to release CL to the OM. These changes would be initiated through the C-terminal region that is composed of a flexible loop and an amphipathic helix. After CL binds within the PbgA cleft, the C-terminal region would associate with the IM and remodel the periplasmic domain, opening up the IM portion of the cleft to the periplasm. Similar to other glycerophospholipids, transport would not require energy in the form of ATP but be achieved through proton motive force and bioenergetically favorable conditions (27).

CL transport from PbgA to the OM can be achieved by either of two means. One way could be through an interacting partner that binds to the cleft of the periplasmic side of PbgA sequestering CL. This unknown protein would then deliver CL to the OM. Another way for CL relocation would be through simple diffusion to the OM directly. PhoPQ interacts with multiple proteins that bridge the periplasmic space, which likely decreases the size of the periplasmic space (28). This reduced distance between the OM and PbgA could facilitate direct interactions that permit the diffusion of CL to the OM (Fig. S6).

10.1128/mBio.03277-19.6 FIG S6 Schematic model of CL transport by PbgA. A schematic model of PbgA-mediated CL transport involves several steps. 1, extraction of CL from the IM glycerophospholipid is accomplished by the tandem Arg215 and Arg216 in the linker region connecting the PbgA TM and periplasmic domains. 2, upon binding of CL to PbgA, CL is loaded into the cleft through the conformational change of the linker region. 3, after binding into the cleft, CL travels through the deep cleft of the protein to the periplasm by a conformational change induced by the C-terminal helix. 4, CL transport to the OM could be through interactions with another partner that binds to the PbgA cleft on the periplasmic side, or CL could also simply diffuse to the OM directly since PhoPQ likely decrease the size of the periplasmic space through multiple interactions of proteins that bridge this space. Download FIG S6, PDF file, 0.3 MB. Copyright © 2020 Fan et al. 2020 Fan et al. This content is distributed under the terms of the Creative Commons Attribution 4.0 International license.

In summary, the crystal structure of PbgA from *S.* Typhimurium reveals the molecular basis of this indispensable membrane protein, including two CL-binding sites, a deep and long cleft that spans the TM and periplasmic domain, a C terminus required for increasing barrier function on the activation of PhoPQ, and a possible catalytic site. This greater understanding of structural details, together with functional studies, provides an important foundation for dissecting how Gram-negative bacteria can increase barrier function in response to host environments.

## MATERIALS AND METHODS

### Protein expression and purification.

The PbgA periplasmic domain (aa 241 to 586) was cloned from the genome of *S.* Typhimurium 14028s into the pET28a vector with a His_6_ tag fused to the C terminus of the coded protein, confirmed using DNA sequencing, and transformed into the E. coli BL21(DE3) strain. The cells were grown at 37°C in LB medium until the cells reached an optical density at 600 nm (OD_600_) of 0.6 to 0.8, and then protein production was induced with 0.5 mM isopropyl-β-d-thiogalactopyranoside (IPTG). After induction at 16°C overnight, cells were harvested by centrifugation at 4,200 × *g*. The cells were resuspended in ice-cold phosphate-buffered saline (PBS) buffer and subjected to two runs of homogenization at 10,000 to 15,000 lb/in^2^ using a French press high-pressure instrument. The homogenate was centrifuged at 17,000 × *g* for 40 min at 4°C, and then the supernatant was collected and loaded on 2 ml of Ni^2+^-nitrilotriacetate (Ni-NTA) affinity resin (Qiagen, Germany) preequilibrated with PBS. After incubating for 1 h, the resin was washed with 50 ml PBS supplemented with 30 mM imidazole. The protein sample was eluted with 10 ml elution buffer containing 500 mM imidazole in PBS and was concentrated to 0.5 ml. The concentrated protein sample was then loaded onto a Superdex-200 column (10/30; GE Healthcare, USA) preequilibrated with 20 mM HEPES (pH 7.0) and 150 mM NaCl. The peak fractions were collected, and the pooled protein sample was concentrated to 30 mg/ml before carrying out crystallization trials. To obtain a seleno-methionine derivative periplasmic protein sample, the E. coli BL21(DE3) strain carrying pET28a-PbgA(241–586) was grown in minimal medium with seleno-l-methionine replacing methionine and purified in the same manner as the native protein.

The full-length PbgA coding sequence (GenBank identifier [ID] 1253750) was cloned from the genome of *S.* Typhimurium 14028s into the pBAD24 vector with a His_6_ tag fused to the C terminus of the coded protein, confirmed using DNA sequencing, and transformed into the E. coli DH5α strain. The cells were grown at 37°C in LB medium until the OD_600_ reached 1.0, and then protein production was induced with 0.2% arabinose. After induction at 37°C for 4 h, the cells were harvested by centrifugation at 4,200 × *g*. The cells were resuspended in ice-cold buffer A (20 mM Tris-HCl [pH 8.0], 150 mM NaCl, 5% [vol/vol] glycerol), and then they were subjected to two runs of homogenization at 10,000 to 15,000 lb/in^2^ using a French press high-pressure instrument. The homogenate was centrifuged at 17,000 × *g* for 10 min at 4°C, and then the supernatant was ultracentrifuged at 100,000 × *g* for 60 min. The membrane fraction was resuspended in buffer A supplemented with 1% (wt/vol) dodecyl-β-d-maltopyranoside (DDM; Anatrace) and was slowly stirred for 1 h at 4°C. After another ultracentrifugation at 100,000 × *g* for 30 min, the supernatant was collected and loaded on 2 ml of Ni-NTA affinity resin (Qiagen, Germany) preequilibrated with buffer A supplemented with 3 mM imidazole and 0.03% (wt/vol) DDM. After 1 h of incubation, the resin was washed with 50 ml buffer A supplemented with 30 mM imidazole and 0.03% (wt/vol) DDM. The protein sample was eluted with 10 ml elution buffer containing buffer A, 500 mM imidazole, and 0.03% (wt/vol) DDM, and the eluted protein was then concentrated to 0.5 ml. The concentrated protein sample was loaded onto a Superdex-200 column (10/30; GE Healthcare, USA) preequilibrated with 20 mM HEPES (pH 7.0), 150 mM NaCl, and 0.03% (wt/vol) DDM. The peak fractions were collected, and the pooled protein sample was concentrated to 20 mg/ml before carrying out crystallization trials. The C-terminal His_6_ tag was left attached. The yield was typically about 1 mg of protein per liter of cell culture.

### Protein crystallization.

Initial periplasmic domain crystallization trials were carried out at room temperature using commercial crystallization screening kits from Hampton Research (USA) by the hanging-drop vapor diffusion method (a 1 μl protein +1 μl well solution drop over a 500 μl reservoir) at 30 mg/ml. Twinning crystals were formed within 6 h. The crystals were optimized by lowering the protein concentration (final concentration, 6 mg/ml), changing the concentration of the precipitant, and using additive screening. Plate-shaped crystals appeared in 6 h and reached full size typically in 2 to 3 days in the presence of a reservoir solution of 0.3 M (NH_4_)_2_SO_4_ and 29% (wt/vol) polyethylene glycol 4000. Se-Met derivative crystals were grown under the same conditions as the native crystals. The crystals were cryo-protected with mother liquor supplemented with 20% (vol/vol) glycerol and flash frozen in liquid nitrogen for storage and data collection.

Before crystallization, purified and concentrated full-length PbgA (20 mg/ml) after fast protein liquid chromatography (FPLC) was mixed with CL at molar ratio of 1:10 on ice for 1 h. Initial crystallization trials were carried out at room temperature using commercial crystallization screening kits from Molecular Dimension (UK) and Hampton Research (USA) by the hanging-drop vapor-diffusion method (a 1 + 1 μl drop over a 500-μl reservoir). Crystals of full-length PbgA grown with DDM diffracted around 3 Å. One of the best crystals gave X-ray diffraction up to 2.7-Å resolution and was used in subsequent data collection. Typically, plate-shaped crystals appeared in 7 days and reached full size (typically 400 by 50 by 20 μm^3^) in 30 days in the presence of a reservoir solution of 0.4 M (NH_4_)_2_SO_4_, 0.1 M morpholineethanesulfonic acid (MES) (pH 6.5), and 10% (wt/vol) polyethylene glycol 3350. The crystals were cryo-protected with mother liquor supplemented with 20% (vol/vol) glycerol and flash-frozen in liquid nitrogen for storage and data collection.

### Data collection and structure determination.

PbgA periplasmic domain (aa 241 to 586) data, seleno-l-methionine derivative PbgA periplasmic domain anomalous data, and the full-length PbgA data were all collected at the Advanced Light Source beamlines. All data were processed using the HKL2000 package (29). For the periplasmic domain structure determination, initial phases were calculated using a SAD data set with the program Phenix.autosol (30). The programs Phenix.autobuild and Coot were used in model building. Refinement was carried out with the phased maximum likelihood method implemented in the program Phenix.refine. Secondary structure restraint was used throughout the refinement. The structure of PbgA was determined by molecular replacement (MR) using the periplasmic domain structure as the search model. For the model building refinement and model validation, the same programs were used as mentioned before. Data collection and structure refinement statistics are summarized in Table S1.

### Ethidium bromide uptake assay.

To validate structural observations and to identify and/or verify putative key residues and regions, we designed a number of mutants using site-directed mutagenesis on plasmid. Then, we generated PCR products and reconstituted them into the *S.* Typhimurium genome using the λ red-mediated gene replacement method (31). OM permeability was then tested by ethidium bromide uptake assay. Bacteria were cultured in LB at 37°C overnight and harvested by centrifugation. The cell pellet was resuspended in PBS at an OD_600_ of 0.2. Carbonyl cyanide *m*-chlorophenylhydrazone (CCCP) was added to halt efflux pump activity for 5 min before adding EtBr to a final concentration of 1.2 μM. Excitation at 530 nm and emission at 600 nm were measured, with readings taken every 5 s for 30 min.

### Infection of BMDMs by *S.* Typhimurium and variants.

The intracellular survival of mutant strains was tested in primary mouse bone marrow-derived macrophages (BMDMs). BMDMs were derived from female BALB/c mice according to standard methods (32). Differentiated macrophages were washed, lifted, and plated into 24-well plates to readhere for 24 h before next-day usage. Bacterial cultures were grown overnight to stationary phase and were diluted in RPMI medium at a multiplicity of infection (MOI) of 20:1 to BMDMs. Bacteria were centrifuged onto BMDM monolayers at 1,000 × *g* for 5 min. Noninternalized bacteria were removed by washing twice with PBS at 30 min postinfection, and any remaining bacteria were killed by adding RPMI medium containing 50 μg/ml gentamicin. At 18 h postinfection, infected macrophages were washed twice with PBS and lysed by adding PBS containing 0.1% Triton X-100 and pipetting up and down. The numbers of intracellular CFU were measured by serial dilution of the samples with PBS and then plating onto LB plates.

### Data availability.

The diffraction data and refined coordinates of the crystal structure of PbgA have been deposited into the PDB under accession code 6V8Q.
