# Supplementary figures and images for: Structure of an Inner Membrane Protein Required for PhoPQ-Regulated Increases in Outer Membrane Cardiolipin
Source: mBio. 2020 Feb 11;11(1):e03277-19. doi: 10.1128/mBio.03277-19 (PMC7018646; doi:10.1128/mBio.03277-19)

Fig S1

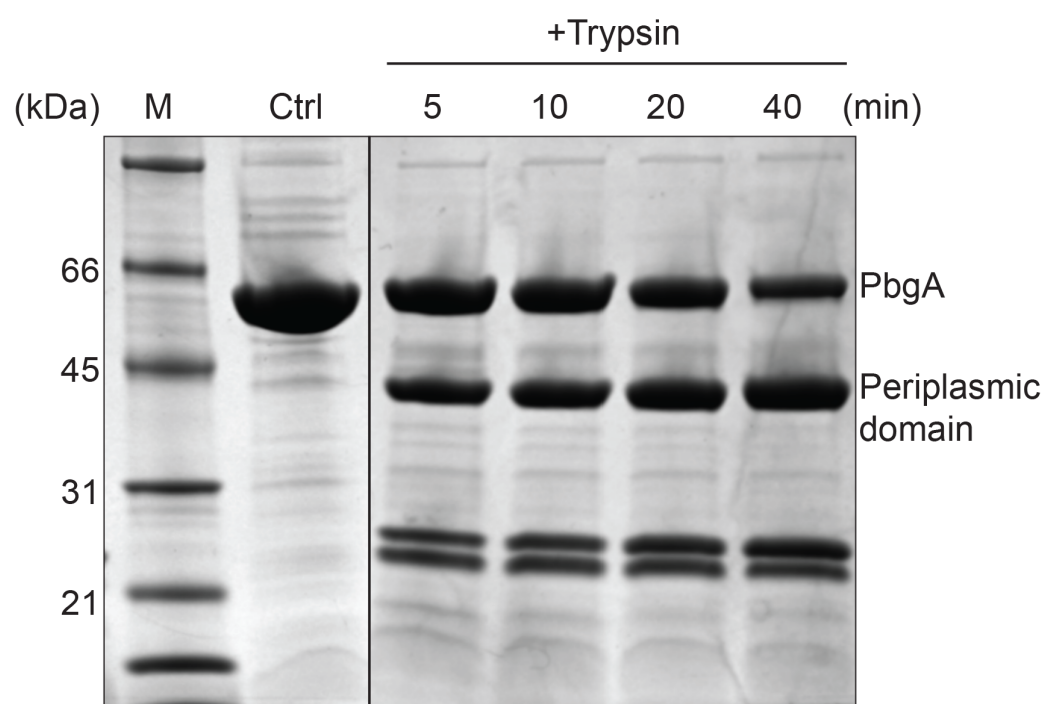

Supplement: FIG S1 [file mBio.03277-19-sf001.pdf]

Fig S2

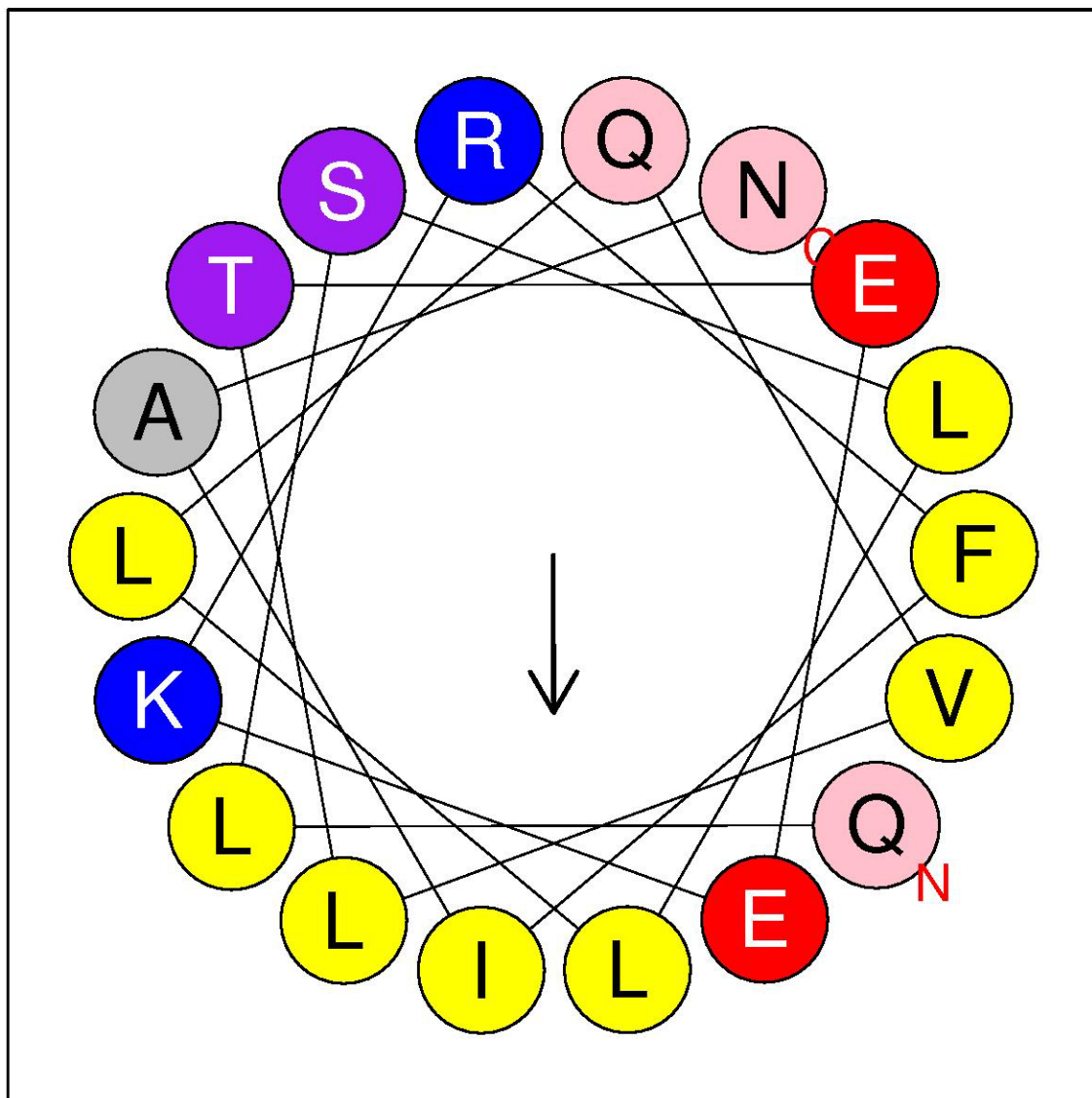

Supplement: FIG S2 [file mBio.03277-19-sf002.pdf]

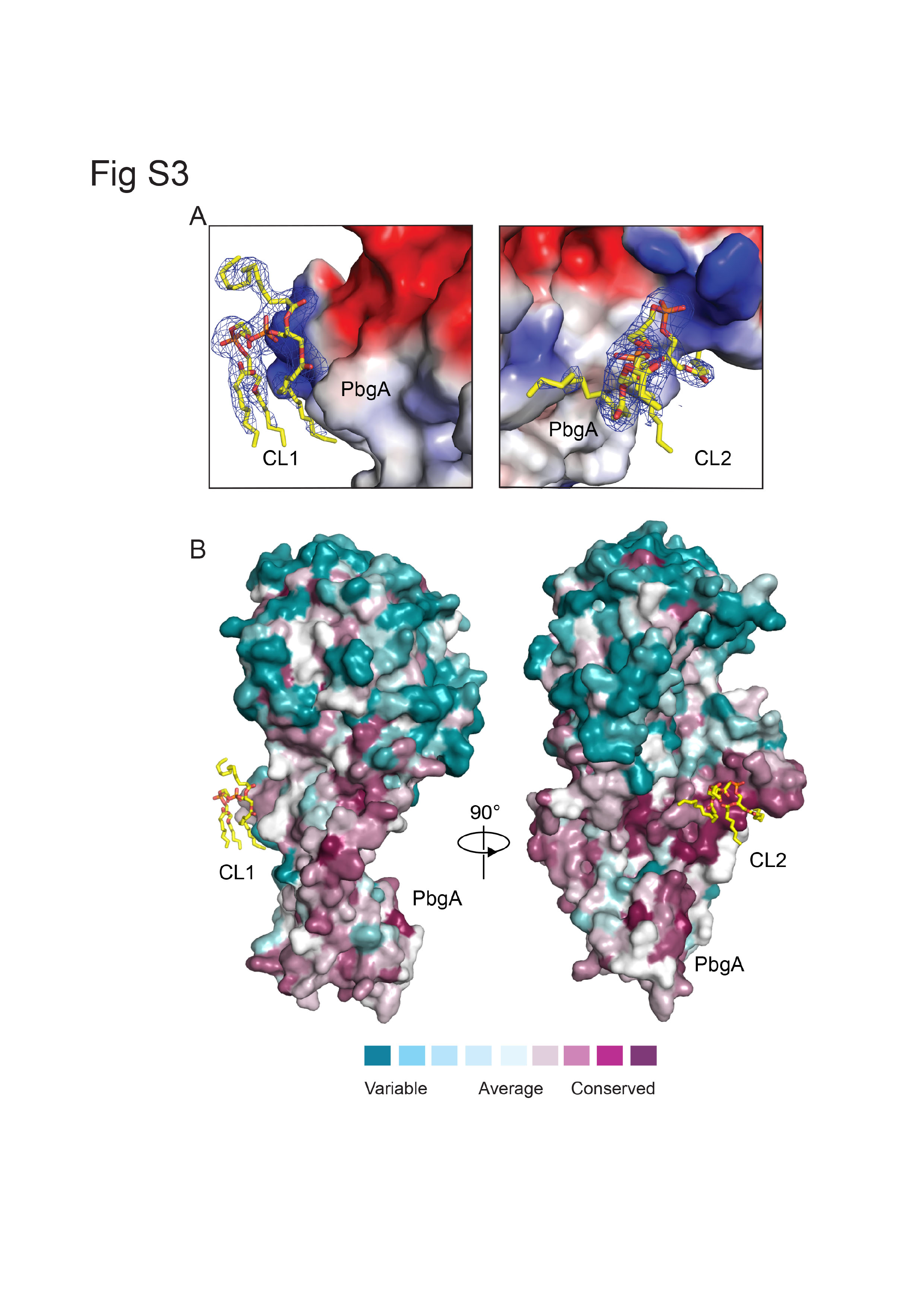

Supplement: FIG S3 [file mBio.03277-19-sf003.jpg]

Fig S5

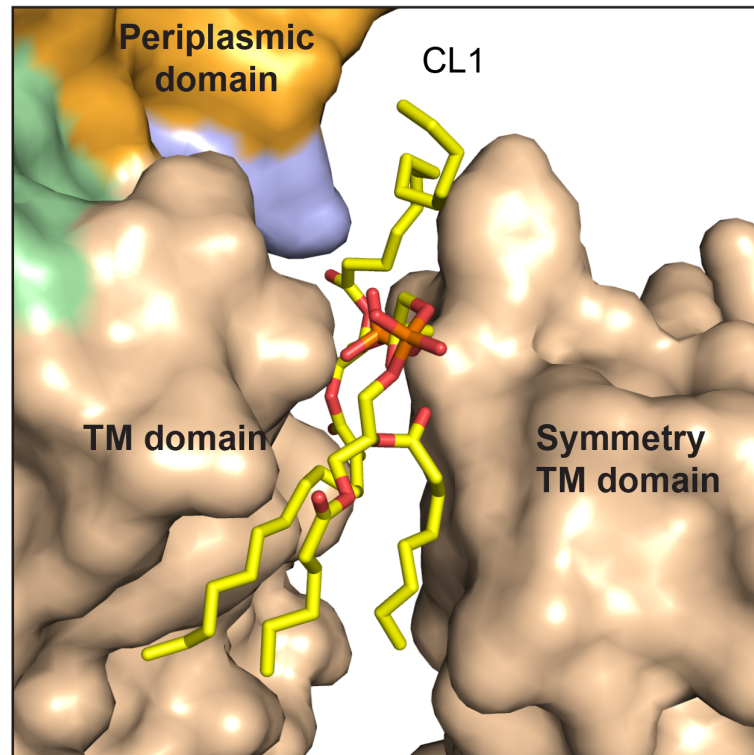

Supplement: FIG S5 [file mBio.03277-19-sf005.pdf]

Fig S6

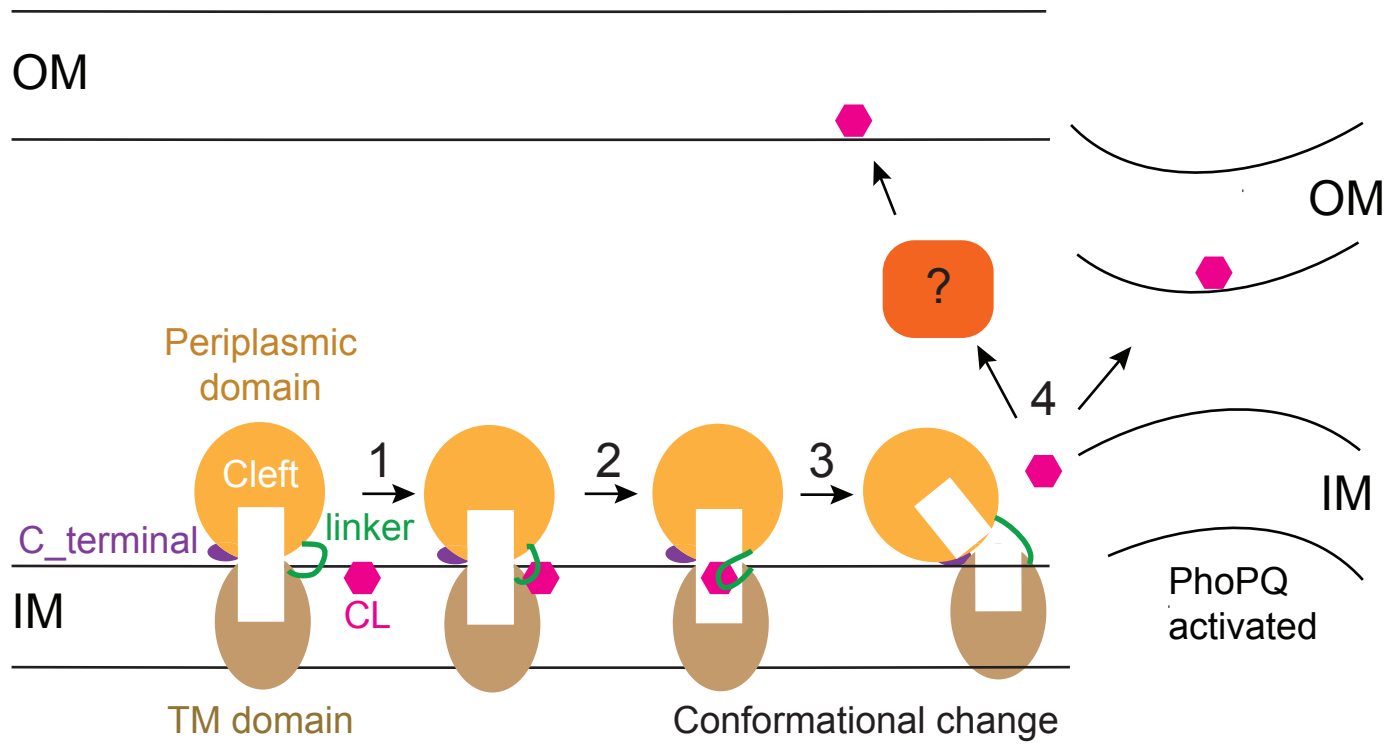

Supplement: FIG S6 [file mBio.03277-19-sf006.pdf]
