# Supplementary material for: Structure of an Inner Membrane Protein Required for PhoPQ-Regulated Increases in Outer Membrane Cardiolipin
Source: mBio. 2020 Feb 11;11(1):e03277-19. doi: 10.1128/mBio.03277-19 (PMC7018646; doi:10.1128/mBio.03277-19)
Supplement: TABLE S1 [file mBio.03277-19-st001.docx]

Data collection and refinement statistics

|  | PbgA | PbgA_PD | PbgA_PD  (Se_SAD) |
| --- | --- | --- | --- |
| **Data collection** |  |  |  |
| Space group | *P2_1_* | *P2_1_* | *P2_1_* |
| Cell dimensions |  |  |  |
| *a, b, c* (Å) | 135.8, 64.8, 146.4 | 51.5, 196.0, 70.6 | 51.5, 194.8, 70.3 |
| *α, β, γ* (°) | 90.0, 97.7, 90.0 | 90.0, 95.8, 90.0 | 90.0, 95.8, 90.0 |
| Wavelength (Å) | 0.99994 | 0.99994 | 0.9790 |
| Resolution (Å) | 50-2.70 (2.80-2.70) ^a^ | 50-1.70 (1.73-1.70) | 50-1.85 (1.92-1.85) |
| Rpim | 8.6 (>100) | 3.7 (44.1) | 4.4 (31.7) |
| *I/σ(I)* | 7.4 (0.6) | 20.3 (1.6) | 13.6 (1.9) |
| Completeness (%) | 97.6 (86.9) | 99.6 (96.7) | 87.2 (79.0) |
| Redundancy | 4.3 (3.5) | 4.2 (3.4) | 5.6 (4.7) |
|  |  |  |  |
| **Refinement** |  |  |  |
| Resolution (Å) | 48.30-2.70 | 42.49-1.70 |  |
| No. reflections | 53782 | 151082 |  |
| *R_work_/ R_free_* | 23.24/29.02 | 19.00/22.80 |  |
| No. atoms |  |  |  |
| Protein | 8978 | 10479 |  |
| Ligand/ion | 286 |  |  |
| B-factors |  |  |  |
| Protein | 52.72 | 21.62 |  |
| Ligand/ion | 70.86 | 28.92 |  |
| R.m.s deviations |  |  |  |
| Bond lengths (Å) | 0.009 | 0.006 |  |
| Bond angles (°) | 1.331 | 1.083 |  |
| Ramachandran plot (%) |  |  |  |
| Favored region | 94.4 | 95.7 |  |
| Allowed region | 5.1 | 3.3 |  |
| Disallowed region | 0.5 | 1.0 |  |

^a^ Values in parentheses are for the highest resolution shell.
