# Supplementary material for: Structure of an Inner Membrane Protein Required for PhoPQ-Regulated Increases in Outer Membrane Cardiolipin
Source: mBio. 2020 Feb 11;11(1):e03277-19. doi: 10.1128/mBio.03277-19 (PMC7018646; doi:10.1128/mBio.03277-19)
Supplement: FIG S4 [file mBio.03277-19-sf004.pdf]

Fig S4

```
PbgA              TM1  TM2
S. Typhimurium    MVTHRQRYREKVSQMVSWGHWFALFNILLATLLGSRYLFVADWPTTLAGRIYSYLSIVGHFSFLV 65
E.coli            MVTHRQRYREKVSQMVSWGHWFALFNILLSLVIGSRYLFIADWPTTLAGRIYSYVSIIGHFSFLV 65
K.pneumoniae      MVTLRQPYREKVSQMVSWGHWFALFNMLLAMVLGSRYLFVADWPTTLAGRLFSYVSLVGHFSFLV 65
P.gergoviae       MVTHRQPYREKVSQMVSWGHWFALFNILLASLLGSRYLFVADWPTTLAGRIYSYISLLGHFSFLV 65
S.blattae         MVTNRQHYREKVSQMISWGHWFALFNILLAIALGSRYLLIADWPTTLAGRIYSWLTLAGHFSFLV 65
Y.pestis          MVTNRQRYREKVSQMISWGHWFALFNILFSLVLGSRYLFVSDWPSSLIGRVYALVSWLGHFSFLV 65
E.billingiae      MVTNRQRYREKVSQMISWGHWFALFNIIFAFILGSRYLFVSDWPASLAGRIYAFTSWMGHFSFIV 65
H.alvei           MVTNRQRYREKVSQMISWGHWFALFNILLGLLLGSRYLFVADWPSSLFGRVYAMISWLGHFSFIG 65
S.glossinidius    MVTKSQRYRDKVSQMISWGHWFALFNILLSLGLGSRYLFISDWPGSLAGRLYAFASWLGHFSFLS 65
D.dadantii        MVTNRPRYREKVSQMISWGHWFALFNILLALGLGSRYLFVADWPSSLAGRVYSLVSWLGHFSFIG 65
```

```
                  TM2  TM3  α1
S. Typhimurium    FATYLLILFPLTFIVMSQRLMRFLSAILATAGMTLLLIDSEVFTRFHLHLNPIVWELVINPDQNE 130
E.coli            FATYLLILFPLTFIVGSQRLMRFLSVILATAGMTLLLIDSEVFTRFHLHLNPIVWQLVINPDENE 130
K.pneumoniae      FTSYVLILFPLTFIVVSQRLMRFLSVILATAGMTLLLIDSEVFTRFHLHLNPVVWELVINPDQNE 130
P.gergoviae       FAVYLLILFPLTFIALSQRLMRFLSVILATAGMTLLLVDSEVFTRFHLHLNPVVWELVINPDQNE 130
S.blattae         FAAYILVLFPLTFVVRSQRLMRFLSVIFATTGQTLLIIDSEVFTRFHLHLNPVVWELVINPDKGE 130
Y.pestis          FAAYLLVIFPLTFVVMSQRLLRFLSAAFATAGLTLLLVDTEVFARFHLHLNPVVWELVVNPEQTE 130
E.billingiae      FAAYLLIVFPLTFVVMSQRLLRFLSAIIATAGLTLILIDSAVFDRFHLHLNPVVWELVVNPDQTE 130
H.alvei           FAAYLLIIFPLTFVVMSQRLLRFLSAALATAGLTLLLVDAEVFTRFHLHLNPVVWELVVNPDQGE 130
S.glossinidius    FTLYLLVVFPLTFVVMSQRLLRLLSAIIATAGLTLLLVDSEVFTRFHQHLNPMVWELVINPYQTE 130
D.dadantii        FSAYLLIIFPLTFIVMSQRLLRFLSAALATTGQTLLIVDAAVFSRFHLHLNMTVWELVTNPEQSE 130
```

```
                  TM4  TM5
S. Typhimurium    MARDWQLMFISVPVILLIEMLFATWSWQKLRSLTRRRHFARPLAAFFFVSFIASHLIYIWADANF 195
E.coli            MARDWQLMFISVPVILLLELVFATWSWQKLRSLTRRRRFARPLAAFLFIAFIASHVVYIWADANF 195
K.pneumoniae      MARDWQLMFISVPVIFLIEMLFATWSWQKLRSLTRRRHYARPVAWFFFLSFVSSHLVYIWADANF 195
P.gergoviae       MARDWQLMFISVPVIFLIEMLMATWSWQKLRSLARRRHYARPVACFLFAAFIATHLMYIWADANF 195
S.blattae         MARDWQLMFICVPVIMLVQLLVASWSWQKLRSLTRRRNYARPVVALFILSFFASHMMYIMADANF 195
Y.pestis          LARDWQLMFISVPVIFLIEMLFGTWAWQKLRSLNRQR-FVKPLVAVLISAFVASHLMYIWADANF 194
E.billingiae      MARDWQLMFISVPAIFLVEMLFATWSWQKLRSLNRRS-FGKPLAVLFIVAFFTSHLMYIWADANF 194
H.alvei           LARDWQLMFICVPVIFLVEMLFGTWSWQKLRSLNRQK-FGKPLAGLFITAFFASHLIYIWADANF 194
S.glossinidius    LARDWQLMFIGIPVIFLVEMLFGTWSWQKLRSLNRYN-IGKPVTALFIGAFCASHVAYIWADANF 194
D.dadantii        MVRDWQLLFISVPVIFMVEMLFGTWCWQKLRSLGRR-HFAKPLAAVFIVAFCLSHLMYIWADANF 194
```

```
                  α2  α3  α4
S. Typhimurium    YRPITMQRANLPLSYPMTARRFLEKHGLLDAQEYQRRLVEQGNPEAVSVQYPLSNLHYRDMGTGQ 260
E.coli            YRPITMQRANLPLSYPMTARRFLEKHGLLDAQEYQRRLIEQGNPDAVSVQYPLSELRYRDMGTGQ 260
K.pneumoniae      YRPITMQRANLPLSYPMTARRFLEKHGLLDAQDYQRRLVEQGAPEAVSVQYPLSNLRYRDLGAGY 260
P.gergoviae       YRPITMQRANLPLSYPMTARRFLEKHGLLNAEEYQRRLVEQGDPEAVSVQYPLSDLRFRDLGTGQ 260
S.blattae         YRPITMQRANLPLSYPMTARRFLEKHGLLNADIYQRRLVEQGNPDAVSVQYPLSELSFRDLGAGY 260
Y.pestis          YRPISMQRANLPLSYPMTARKFLEKHGLLDQQEYQRRLTEQGNPDALAVEYPLNPITFNDKGSGY 259
E.billingiae      YRPITMQRSNLPLSYPMTARRFLEKHGLLDAQEYQRRLVQQGNPEAVSVAYPLNDITFRDKGTGS 259
H.alvei           YRPITMQRSNLPLSYPMTARRFLEKHGLLDAQEFQNKLTQTGSPEAVSVEYPLNKLSFADKGAGY 259
S.glossinidius    YRPITMQRANLPLSYPMTARRFLERHGLLDAQAFERRLVQQGNPEAAAVEYPLSPVSFSDKGSGL 259
D.dadantii        YRPITMQRANLPLSYPMTARRFLEKHGLLNAQEYERRLTLQGNPDAVSVEYPLSDINFRDAGSGY 259
```

```
                  β1  α5  α6  β2  α7  α8
S. Typhimurium    NVLLITVDGLNYSRFEKQMPELATFAEQNIDFTRHMSSGNTTDNGIFGLFYGISPGYMDGVLSTR 325
E.coli            NVLLITVDGLNYSRFEKQMPALAGFAEQNISFTRHMSSGNTTDNGIFGLFYGISPSYMDGILSTR 325
K.pneumoniae      NVLLITVDNLNYSRFEKTMPALAAFAKENVNFTQHMSSGNTADSGLFGLFYGISPGYMDGVLSAR 325
P.gergoviae       NVLLITVDGLNYSRFEKQMPKLADFAQQNINFTQHMSAGNDTDSGIFGLFYGISPAYTDGILSTR 325
S.blattae         NVLLITVDGLNYSRFTETMPALARFASQNASFTQHMSAGNNTDNGIFGLFYGISPSYMDGILATR 325
Y.pestis          NLLLIVVDGIRVNSLQQDMPALAEFGRENIQFDHHYSSGNRQDTGLFGLFYGISPTYWDGILSAR 324
E.billingiae      NLLIITVDGLNESTIAKSLPHLAQFAQQNVRFTQHYSSGNNADSGLFGLFYGISASYMDGVLSNR 324
H.alvei           NLLVIAVDGLRADDLKQDLPQLAAFGNDNLEFTQHYSSGNNLDNGLFGLFYGISPSYLEGILAGR 324
S.glossinidius    NLLIITTGMLRNDTLDQQMPALKRFAEQNARFAQHYSTGNQLDTGLFGLFYGISASYLDGILATR 324
D.dadantii        NLLLVVMDSVPDQDTPRSMPNLARFAEENVRFSNHFSAGTQQDGGLFNLFYGISTTYMDGVLGSS 324
```

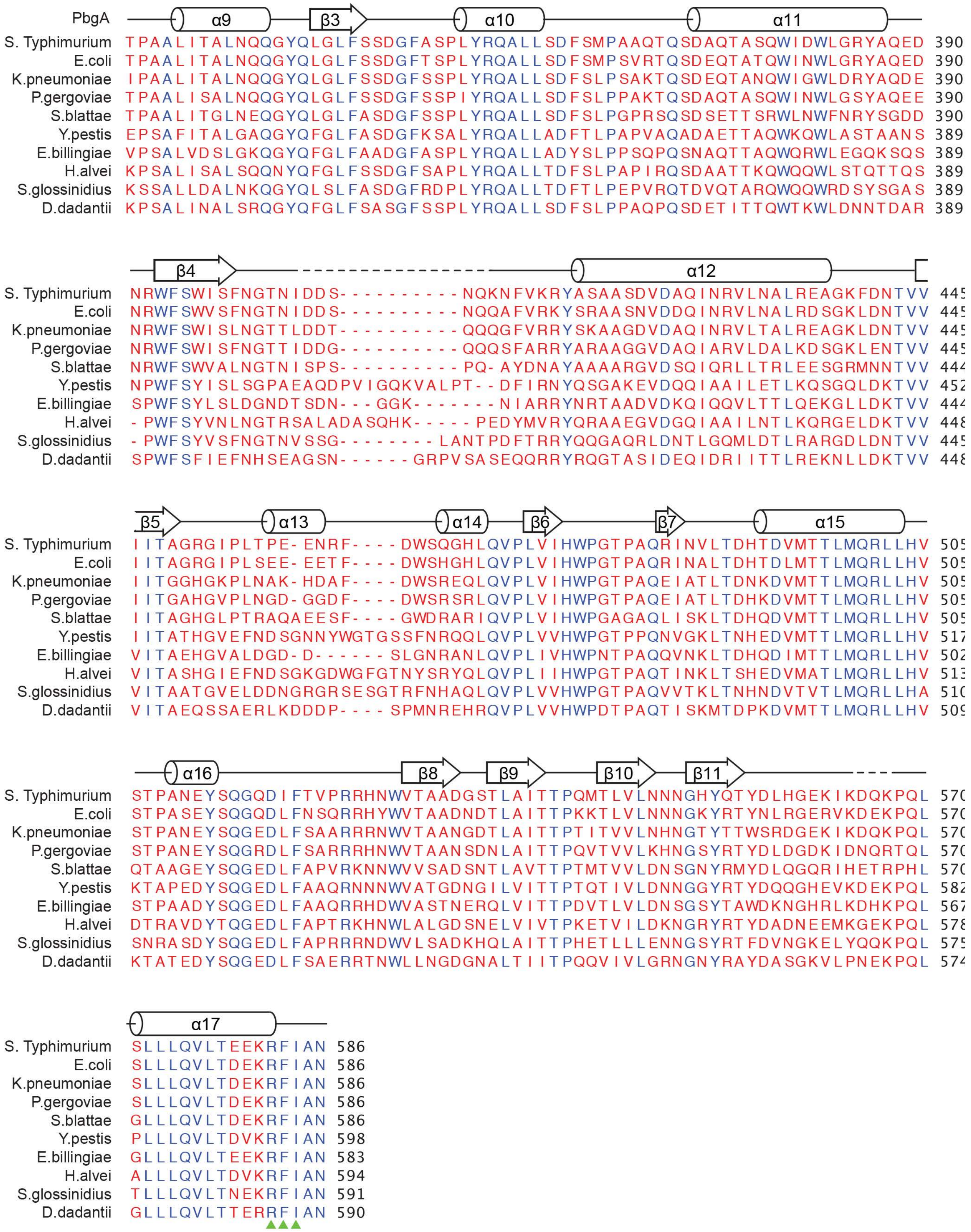
PbgA
α9 β3 α10 α11
S. Typhimurium TPAALITALNQQGYQLGLFSSDGFASPLYRQALLSDFSMPAAQTQSDAQTASQWIDWLGRYAQED 390
E.coli TPAALITALNQQGYQLGLFSSDGFTSPLYRQALLSDFSMPSVRTQSDEQTATQWINWLGRYAQED 390
K.pneumoniae IPAALITALNQQGYQLGLFSSDGFSSPLYRQALLSDFSLPSAKTQSDEQTANQWIGWLDRYAQDE 390
P.gergoviae TPAALISALNQQGYQLGLFSSDGFSSPIYRQALLSDFSLPPAKTQSDAQTASQWINWLGSYAQEE 390
S.blattae TPAALITGLNEQGYQLGLFASDGFSSPLYRQALLSDFSLPGPRSQSDSETTSRWLNWFNRYSGDD 390
Y.pestis EPSAFITALGAQGYQFGLFASDGFKSALYRQALLADFTLPAPVAQADAETTAQWKQWLASTAANS 389
E.billingiae VPSALVDSLGKQGYQFGLFAADGFASPLYRQALLADYSLPPSQPQSNAQTTAQWQRWLEGQKSQS 389
H.alvei KPSALISALSQQNYQFGLFSSDGFSAPLYRQALLTDFSLPAPIRQSDAATTKQWQQWLSTQTTQS 389
S.glossinidius KSSALLDALNKQGYQLSLFASDGFRDPLYRQALLTDFTLPEPVRQTDVQTARQWQQWRDSYSGAS 389
D.dadantii KPSALINALSRQGYQFGLFSASGFSSPLYRQALLSDFSLPPAQPQSDETITTQWTKWLDNNTDAR 389
β4 α12
S. Typhimurium NRWFSWISFNGTNIDDS-----------NQKNFVKRYASAASDVDAQINRVLNALREAGKFDNTVV 445
E.coli NRWFSWVSFNGTNIDDS-----------NQQAFVRKYSRAASNVDDQINRVLNALRDSGKLDNTVV 445
K.pneumoniae NRWFSWISLNGTTLDDT-----------QQQGFVRRYSKAAGDVDAQINRVLTALREAGKLDNTVV 445
P.gergoviae NRWFSWISFNGTTIDDG-----------QQQSFARRYARAAGGVDAQIARVLDALKDSGKLENTVV 445
S.blattae NRWFSWVALNGTNISPS-----------PQ-AYDNAYAAAARGVDSQIQRLLTRLEESGRMNNTVV 444
Y.pestis NPWFSYISLSGPAEAQDPVIGQKVALPT--DFIRNYQSGAKEVDQQIAAILETLKQSGQLDKTVV 452
E.billingiae SPWFSYLSLDGNDTSDN---GGK--------NIARRYNRTAADVDKQIQQVLTTLQEKGLLDKTVV 444
H.alvei -PWFSYVNLNGTRSALADASQHK-----PEDYMVRYQRAAEGVDGQIAAILNTLKQRGELDKTVV 448
S.glossinidius -PWFSYVSFNGTNVSSG---------LANTPDFTRRYQQGAQRLDNTLGQMLDTLRARGDLDNTVV 445
D.dadantii SPWFSFIEFNHSEAGSN------GRPVSASEQQRRYRQGTASIDEQIDRIITTLREKNLLDKTVV 448
β5 α13 α14 β6 β7 α15
S. Typhimurium IITAGRGIPLTPE-ENRF----DWSQGHLQVPLVIHWPGTPAQRINVLTDHTDVMTTLMQRLLHV 505
E.coli IITAGRGIPLSEE-EETF----DWSHGHLQVPLVIHWPGTPAQRINALTDHTDLMTTLMQRLLHV 505
K.pneumoniae IITGGHGKPLNAK-HDAF----DWSREQLQVPLVIHWPGTPAQEIATLTDNKDVMTTLMQRLLHV 505
P.gergoviae IITGAHGVPLNGD-GGDF----DWSRSRLQVPLVIHWPGTPAQEIATLTDHKDVMTTLMQRLLHV 505
S.blattae IITAGHGLPTRAQAEESF----GWDRARIQVPLVIHWPGAGAQLISKLTDHQDVMTTLMQRLLHV 505
Y.pestis IITATHGVEFNDSGNNYWGTGSSFNRQQLQVPLVVHWPGTPPQNVGKLTNHEDVMTTLMQRLLHV 517
E.billingiae VITAEHGVALDGD-D-------SLGNRANLQVPLIVHWPNTPAQQVNKLTDHQDIMTTLMQRLLHV 502
H.alvei VITASHGIEFNDSGKGDWGFGTNYSRYQLQVPLIIHWPGTPAQTINKLTSHEDVMATLMQRLLHV 513
S.glossinidius VITAATGVELDDNGRGRSESGTRFNHAQLQVPLVVHWPGTPAQVVTKLTNHNDVTVTLMQRLLHA 510
D.dadantii VITAEQSSAERLKDDDP----SPMNREHRQVPLVVHWPDTPAQTISKMTDPKDVMTTLMQRLLHV 509
α16 β8 β9 β10 β11
S. Typhimurium STPANEYSQGQDIFTVPRRHNWVTAADGSTLAITTPQMTLVLNNNGHYQTYDLHGEKIKDQKPQL 570
E.coli STPASEYSQGQDLFNSQRRHYWVTAADNDTLAITTPKKTLVLNNNGKYRTYNLRGERVKDEKPQL 570
K.pneumoniae STPANEYSQGEDLFSAARRRNWVTAANGDTLAITTPTITVVLNHNGTYTTWSRDGEKIKDQKPQL 570
P.gergoviae STPANEYSQGRDLFSARRRHNWVTAANSDNLAITTPQVTVVLKHNGSYRTYDLDGDKIDNQRTQL 570
S.blattae QTAAGEYSQGEDLFAPVRKNNWVVSADSNTLAVTTPTMTVVLDNSGNYRMYDLQGQRIHETRPHL 570
Y.pestis KTAPEDYSQGEDLFAAQRNNNWVATGDNGILVITTPTQTIVLDNNGGYRTYDQQGHEVKDEKPQL 582
E.billingiae STPAADYSQGEDLFAAQRRHDWVASTNERQLVITTPDVTLVLDNSGSYTAWDKNGHRLKDHKPQL 567
H.alvei DTRAVDYTQGEDLFAPTRKHNWLALGDSNELVIVTPKETVILDKNGRYRTYDADNEEMKGEKPQL 578
S.glossinidius SNRASDYSQGEDLFAPRRRNDWVLSADKHQLAITTPHETLLLENNGSYRTFDVNGKELYQQKPQL 575
D.dadantii KTATEDYSQGEDLFSAERRTNWLLNGDGNALTIITPQQVIVLGRNGNYRAYDASGKVLPNEKPQL 574
α17
S. Typhimurium SLLLQVLTEEKRFIAN 586
E.coli SLLLQVLTDEKRFIAN 586
K.pneumoniae SLLLQVLTDEKRFIAN 586
P.gergoviae SLLLQVLTDEKRFIAN 586
S.blattae GLLLQVLTDEKRFIAN 586
Y.pestis PLLLQVLTDVKRFIAN 598
E.billingiae GLLLQVLTEEKRFIAN 583
H.alvei ALLLQVLTDVKRFIAN 594
S.glossinidius TLLLQVLTNEKRFIAN 591
D.dadantii GLLLQVLTTERRFIAN 590
